# Severe Hypophosphatemia Occurring After Repeated Exposure to a Parenteral Iron Formulation

**DOI:** 10.1155/2022/1011401

**Published:** 2022-10-07

**Authors:** Keerthana Haridas, Alice Yau

**Affiliations:** ^1^Internal Medicine Icahn School of Medicine, Mount Sinai St Luke's/West, New York, NY, USA; ^2^Endocrinology Icahn School of Medicine, Mount Sinai, Beth, Israel

## Abstract

Hypophosphatemia is a less known complication of parenteral iron use, particularly after the use of certain iron formulations. We report the case of a young male with inflammatory bowel disease and iron deficiency anemia, who developed severe symptomatic hypophosphatemia after his third exposure to iron carboxymaltose with no evidence of the same occurring upon prior exposures to the compound. Investigations revealed serum phosphorous levels of 0.7 mg/dl, corrected serum calcium of 8–9.5 mg/dl, alkaline phosphatase of 50 U/L (38–126), 25 hydroxy vitamin D level of 40.2 ng/ml, and intact PTH elevated to 207 pg/ml. Urine studies indicated renal phosphate wasting. Presentation was not in keeping with refeeding syndrome. Intact fibroblast growth factor 23 level, measured after the initiation of treatment was within the normal range at 179 RU/mL (44–215). 1,25 dihydroxy vitamin D level, also measured after the initiation of treatment, was normal at 26.3 pg/ml (19.9–79.3). The patient was treated with calcitriol and aggressive oral and intravenous phosphorous repletion. Symptoms then resolved and the patient was discharged on an oral regimen. This phenomenon is postulated to occur due to an increase in the level and activity of FGF23 and decreased cleavage of the same, due to anemia as well as use of specific iron formulations. This is the first instance, in our literature review, of this complication known to occur, not after initial exposure to an implicated iron formulation but occurring on subsequent exposure.

## 1. Introduction

Hypophosphatemia is a lesser-known side effect of intravenous iron repletion, particularly with certain formulations of iron including saccharated ferric oxide and ferric carboxymaltose [1]. First described in the late 1980s [2] with the former, it has since been reported in more cases, induced by the latter [1] although clear timelines associated with development, time to nadir, and duration to resolution are less clear. While it is transient and self-limiting in most cases, it can be symptomatic and long lasting, requiring aggressive treatment and supplementation in other instances [3–5].

## 2. Case Report

A 35 year old male with a past medical history of Crohn's disease with associated malnutrition (diagnosed 20 years ago, refractory to therapy with multiple medical agents and with no prior history of surgery, on methotrexate, Ustekinumab and prednisone) as well as iron deficiency anemia (mean Hgb in the range of 6–9 gdl, serum Iron 9–29 mg/dl, total iron binding capacity-286–303 mcg/dl, transferrin receptor saturation 3–10%, and ferritin 3–73 mg/ml) was admitted after the detection of severe hypophosphatemia (serum phosphorus-0.7 mg/dl), two weeks after receiving 685 mg of parenteral ferric carboxymaltose. Patient's treatment for Crohn's disease had been started 4 years ago. The patient reported no symptoms at presentation but endorsed minimal tingling and cramping in fingers of bilateral upper extremities two days after presentation. No other sensorimotor complaints were endorsed. No cardiovascular or respiratory symptoms were endorsed. The patient reported no change in appetite or diet or no recent alteration in food intake, endorsed good oral intake and no recent weight loss. General examination was notable for Body Mass Index (BMI) of 16.14 kg/m^2^. Examination was unremarkable and no focal sensorimotor deficits were noted. Chvostek's and Trousseau's signs were negative.

Investigations revealed serum phosphorous levels of 0.7 mg/dl, corrected serum calcium of 8–8.5 mg/dl which rose to 9–9.5 mg/dl after Vitamin D supplementation was started, alkaline phosphatase of 50 U/L (38–126), with bone specific alkaline phosphatase level, measured using ELISA, of 8.5 mcg/L (4–27). 25 hydroxy vitamin D level measured using ELISA was 40.2 ng/ml, and intact PTH was elevated to 207 pg/ml. 24 hour urine phosphorous level was 1.2 g and TMP/GFR (tubular maximum reabsorption of phosphate per glomerular filtration rate) was 1.39 mg/dl (normal range 2.6–3.8) with fractional tubular excretion of phosphate of 31.82%, suggestive of renal phosphate wasting.

1,25 dihydroxy vitamin D level, measured by RIA, was 26.3 pg/ml (19.9–79.3); although, this was measured after calcitriol supplementation was initiated. Intact fibroblast growth factor 23 level was measured using ELISA to be 179 RU/mL (44–215) although the level was obtained 8 days after initiation of treatment.

Interestingly, the patient had received infusions of the same formulation in doses of 675 mg and 700 mg in 2018 and 750 mg in 2017 with no documented evidence of consequent hypophosphatemia (Serum P 3.3–3.7 mg/dl). The patient also received iron dextran and ferrous gluconate in the 2 months prior to the described instance with no resulting hypophosphatemia.

The patient was started on calcitriol 0.25 mcg daily which was then increased to 0.5 mcg daily to achieve phosphate repletion while ensuring normalized serum calcium levels. Corrected serum calcium levels rose to 9–9.5 mg/dl after this supplementation was started. PTH level, which was likely elevated secondary to mild hypocalcemia, was slated to be monitored upon outpatient follow up.

Aggressive phosphate supplementation was initiated through oral and parenteral routes due to severity of hypophosphatemia and likely coexistent malabsorption in the setting of Crohn's disease. Symptoms subsided after repletion was started.

The patient's phosphorous level rose to a maximum of 2.2 mg/dl during the hospitalization (day 24 post infusion) and the patient was discharged on sodium phosphate 500 mg four times daily and calcitriol 0.5 mcg daily with close follow-up scheduled.

## 3. Discussion

Hypophosphatemia has been increasingly reported following therapy with parenteral iron formulations recently [6]. Certain formulations like ferric carboxymaltose and saccharated ferric oxide are implicated at a higher frequency than others [1, 4, 6]. This patient developed hypophosphatemia after his second exposure to Ferric carboxymaltose, although he did not develop the same after his first exposure. This has not been reported in any instances, in our literature review. It is unclear why this occurred as the patient had no flares of inflammatory bowel disease, altered oral intake or exposure to higher doses of the iron formulation.

The occurrence of de novo hypophosphatemia is reported with varying incidences across studies, but most studies reported an incidence between 18.5% and 50% with about a third to half of patients developing severe hypophosphatemia (*P* < 1 mg/dl) [6–8]. Severe acute hypophosphatemia can result in muscle cramps and spasms, rhabdomyolysis, cardiac arrhythmias and conduction disturbances, while chronic hypophosphatemia may present as nonspecific fatigue, bone pain, osteomalacia and pathological fractures [6].

The presence of coexistent inflammatory bowel disease or other causes of malabsorption, low body weight, and African American race, as seen in our patient have been shown to increase the risk of developing hypophosphatemia [6–8].

The refeeding syndrome was ruled out as the patient had no prior history of decrease followed by increase in dietary intake, no other electrolyte abnormalities, no episodes of hypoglycemia and hypophosphatemia resolved with no alteration in feeding rate while in the hospital. In addition, Ustekinumab has not been associated with hypophosphatemia.

Hypophosphatemia is caused due to an increase in the amount of intact fibroblast growth factor 23 (FGF 23) in circulation [1, 6]. FGF 23 is largely produced by the bone and acts on its receptors in the renal tubules where it inhibits the sodium-phosphorus cotransporters (NaPi-2a and NaPi-2c) on the apical membrane of proximal tubule cells [9]. It also decreases the synthesis of 1,25 dihydroxy vitamin D, thus lowering calcium and phosphate absorption and reabsorption, consequently decreasing their levels in circulation [9]. It also increases calcium reabsorption through the TRVP5 (transient receptor potential cation channel subfamily V member 5) channel, thus decreasing the occurrence of hypocalcemia due to coexistent vitamin D deficiency [9].

Iron deficiency and the resulting anemia increase the levels of iFGF23 through an increase in the level of hypoxia inducible factor alpha 1 (HIF-*α*1) [9]. However, it also increases cleavage of iFGF23 in osteocytes, resulting in an increase in the level of C terminal FGF23 (cFGF23), which inhibits the action of iFGF23 [9, 10]. Ferric carboxymaltose, however, decreases the cleavage of iFGF23, increasing levels of the bioactive precursor [9, 10]. In addition, an increase in the uptake of phosphate intracellularly during erythropoiesis contributes to a drop in measured value in the serum [9]. The level of iFGF23 in our patient was normal, most likely as it was measured after the initiation of treatment and improvement in hypophosphatemia. The level of cFGF23 unfortunately, could not be measured in our patient.

The timeline for development of hypophosphatemia indicates that the lowest levels of serum phosphorus were noted at week 2 and increased in the following weeks [6]. The time to resolution varied between a mean of 8 to 12 weeks but certain instances of persistent hypophosphatemia that lasted as long as 6 months to 2 years have been reported [6, 10, 11]. Parathyroid hormone levels are usually elevated given the low 1,25 hydroxy vitamin D level, as in our patient. Patients are treated with calcitriol and aggressive phosphate repletion as needed. Studies however report minimal utility in treating moderately low phosphorus levels unless severe hypophosphatemia (<1 mg/dl) is reported or the patient is symptomatic [12]. One report also described success with the use of Burosumab, a monoclonal antibody directed against FGF23 in a patient with severe iron‐induced osteomalacia [13].

## 4. Conclusion

Hypophosphatemia is a little known but common adverse effect with the use of certain preparations of intravenous iron, especially in patients with antecedent risk factors, must be anticipated and treated in order to avoid osseous and systemic complications. In addition, this may develop upon subsequent exposures to a particular intravenous formulation even if not encountered after first exposure, like in our patient, and must therefore not be ruled out in these situations.

## Data Availability

No data were used to support this study.

## Additional Points

*List of assays used in case report*. (i) 25 hydroxy Vitamin D level: Measured by LIAISON® 25 OH Vitamin D TOTAL Assay antibody-AB_2811287. Direct Competitive immunoluminometric assay using coated magnetic microparticles. Kit contains: magnetic particles coated with a goat antibody directed against 25-OH vitamin D. (ii) 1,25 dihydroxy Vitamin D: Measured by 1,25 Dihydroxy Vitamin D RIA Kit-AB_2895011. The identity of the antibodies in this kit have not been verified and kit contents can vary-use with caution. (iii) Bone specific alkaline phosphatase-Measured by ELISA-AB_959050. ELISA/Western blot. (iv) FGF32-Measured using Immunotropics FGF 23 (C-Term) ELISA kit-AB_2722648. ELISA Kit contains: two affinity purified goat polyclonal antibodies selected to detect epitopes within the carboxyl-terminal (C-Term) portion of FGF-23. One antibody is biotinylated for capture and the other antibody is conjugated with the enzyme horseradish peroxidase (HRP) for detection. These antibodies bind to both the intact molecule and large carboxyl-terminal fragments of human FGF-23.

## References

[1] Zoller H., Schaefer B., Glodny B. (2017). Iron-induced hypophosphatemia: an emerging complication. *Current Opinion in Nephrology and Hypertension*.

[2] Okada M., Imamura K., Fuchigami T. (1982). 2 cases of nonspecific multiple ulcers of the small intestine associated with osteomalacia caused by long-term intravenous administration of saccharated ferric oxide. *Nihon Naika Gakkai Zasshi*.

[3] Bartko J., Roschger P., Zandieh S., Brehm A., Zwerina J., Klaushofer K. (2018). Hypophosphatemia, severe bone pain, gait disturbance, and fatigue fractures after iron substitution in inflammatory bowel disease: a case report. *Journal of Bone and Mineral Research*.

[4] Bishay R. H., Ganda K., Seibel M. J. (2017). Long-term iron polymaltose infusions associated with hypophosphataemic osteomalacia: a report of two cases and review of the literature. *Therapeutic Advances in Endocrinology and Metabolism*.

[5] Fang W., McMahon L. P., Bloom S., Garg M. (2019). Symptomatic severe hypophosphatemia after intravenous ferric carboxymaltose. *JGH Open*.

[6] Glaspy J. A., Lim-Watson M. Z., Libre M. A. (2020). Hypophosphatemia associated with intravenous iron therapies for iron deficiency anemia: a systematic literature review. *Therapeutics and Clinical Risk Management*.

[7] Schaefer B., Würtinger P., Finkenstedt A. (2016). Choice of high-dose intravenous iron preparation determines hypophosphatemia risk. *PLoS One*.

[8] Schaefer B., Tobiasch M., Viveiros A. (2021). Hypophosphataemia after treatment of iron deficiency with intravenous ferric carboxymaltose or iron isomaltoside-a systematic review and meta-analysis. *British Journal of Clinical Pharmacology*.

[9] Coppolino G., Nicotera R., Cernaro V. (2020). Iron infusion and induced hypophosphatemia: the role of fibroblast growth factor-23. *Therapeutic Apheresis and Dialysis*.

[10] Blazevic A., Hunze J., Boots J. M. M. (2014). Severe hypophosphataemia after intravenous iron administration. *The Netherlands Journal of Medicine*.

[11] Glaspy J. A., Wolf M., Strauss W. E. (2021). Intravenous iron-induced hypophosphatemia: an emerging syndrome. *Advances in Therapy*.

[12] García Martín A., Varsavsky M., Cortés Berdonces M. (2020). Phosphate disorders and clinical management of hypophosphatemia and hyperphosphatemia. *Endocrinología, Diabetes y Nutrición*.

[13] Amarnani R., Travis S., Javaid M. K. (2020). Novel use of burosumab in refractory iron-induced FGF23-mediated hypophosphataemic osteomalacia. *Rheumatology*.

